# Deep learning reconstruction algorithm and high-concentration contrast medium: feasibility of a double-low protocol in coronary computed tomography angiography

**DOI:** 10.1007/s00330-024-11059-x

**Published:** 2024-09-19

**Authors:** Damiano Caruso, Domenico De Santis, Giuseppe Tremamunno, Curzio Santangeli, Tiziano Polidori, Giovanna G. Bona, Marta Zerunian, Antonella Del Gaudio, Luca Pugliese, Andrea Laghi

**Affiliations:** Department of Medical Surgical Sciences and Translational Medicine, Sapienza University of Rome, Rome, Italy

**Keywords:** Contrast media, Computed tomography angiography, Deep learning, Image processing, Radiation dosage

## Abstract

**Objective:**

To evaluate radiation dose and image quality of a double-low CCTA protocol reconstructed utilizing high-strength deep learning image reconstructions (DLIR-H) compared to standard adaptive statistical iterative reconstruction (ASiR-V) protocol in non-obese patients.

**Materials and methods:**

From June to October 2022, consecutive patients, undergoing clinically indicated CCTA, with BMI < 30 kg/m^2^ were prospectively included and randomly assigned into three groups: group A (100 kVp, ASiR-V 50%, iodine delivery rate [IDR] = 1.8 g/s), group B (80 kVp, DLIR-H, IDR = 1.4 g/s), and group C (80 kVp, DLIR-H, IDR = 1.2 g/s). High-concentration contrast medium was administered. Image quality analysis was evaluated by two radiologists. Radiation and contrast dose, and objective and subjective image quality were compared across the three groups.

**Results:**

The final population consisted of 255 patients (64 ± 10 years, 161 men), 85 per group. Group B yielded 42% radiation dose reduction (2.36 ± 0.9 mSv) compared to group A (4.07 ± 1.2 mSv; *p* < 0.001) and achieved a higher signal-to-noise ratio (30.5 ± 11.5), contrast-to-noise-ratio (27.8 ± 11), and subjective image quality (Likert scale score: 4, interquartile range: 3–4) compared to group A and group C (all *p* ≤ 0.001). Contrast medium dose in group C (44.8 ± 4.4 mL) was lower than group A (57.7 ± 6.2 mL) and B (50.4 ± 4.3 mL), all the comparisons were statistically different (all *p* < 0.001).

**Conclusion:**

DLIR-H combined with 80-kVp CCTA with an IDR 1.4 significantly reduces radiation and contrast medium exposure while improving image quality compared to conventional 100-kVp with 1.8 IDR protocol in non-obese patients.

**Clinical relevance statement:**

Low radiation and low contrast medium dose coronary CT angiography protocol is feasible with high-strength deep learning reconstruction and high-concentration contrast medium without compromising image quality.

**Key Points:**

- *Minimizing the radiation and contrast medium dose while maintaining CT image quality is highly desirable*.
- *High-strength deep learning iterative reconstruction protocol yielded 42% radiation dose reduction compared to conventional protocol*.
- *“Double-low” coronary CTA is feasible with high-strength deep learning reconstruction without compromising image quality in non-obese patients*.

## Introduction

Coronary computed tomography angiography (CCTA) is advised as the primary diagnostic test in individuals exhibiting low-to-intermediate risk of obstructive coronary artery disease (CAD) when clinical evaluation is not conclusive and as a first-line test in patients without known CAD and stable chest pain, according to ESC 2019 [1] and SCCT 2021 [2] guidelines, respectively, attributed to its notable high negative predictive value.

According to the PROTECTION VI multicenter study, radiation exposure associated with cardiovascular CT has been reduced by 78% from 2007 to 2017 [3]. Nevertheless, the number of CCTA has rapidly increased over the last decade and the cumulative radiation exposure during CCTA is still a major concern related to long-term carcinogenesis [4]. In this regard, the amount of iodinated contrast medium is not only is risk factor for contrast-induced acute kidney injury but also plays a role in amplifying radiation-induced DNA damage [5, 6].

Under this perspective, the “double-low dose” strategy, consisting of reducing both radiation output and contrast medium volume, represents an enticing research field. For this purpose, a high-concentration contrast medium helps reduce the total volume of the administered contrast medium and facilitates lowering the radiation output [7, 8]. Nevertheless, low tube voltages lead to heightened image noise attributed to the diminished penetrating capability of low-energy X-rays, especially in patients with high body mass index (BMI) [9]. This drawback was potentially counterbalanced by iterative reconstruction (ASiR-V) algorithms, consisting of statistical models designed to reduce image noise through several iterations of the reconstruction process. Despite effectively enabling low-dose examinations with acceptable levels of image noise, this technology ultimately leads to alterations in image texture, resulting in overly smoothed images when fully exploited. Deep learning image reconstruction (DLIR) algorithms based on deep convolutional neural networks have been recently introduced. These advanced algorithms, trained on large low-dose data, are designed to learn specific characteristics of noise and artifacts and to remove them minimizing detrimental effects on image quality. Hence, they promise shorter reconstruction times and significantly reduced noise levels while maintaining the integrity of image texture [10–13]. High-strength deep learning image reconstructions (DLIR-H) have been recently proven effective in reducing radiation and contrast medium dose in CCTA compared to ASiR-V in normal-size patients [14]. However, no definitive data are available on DLIR-H performances in a broader patient cohort consisting also of overweight patients.

Therefore, the aim of our study is to evaluate the radiation dose and image quality of a double-low CCTA protocol reconstructed with DLIR-H in comparison with standard-dose ASIR-V protocol in a cohort of non-obese patients.

## Materials and methods

### Patient population

This prospective single-center study received approval from the local institutional review board, and written informed consent was obtained from all participants. Consecutive individuals who underwent clinically indicated CCTA for either known or suspected CAD were enrolled from June to December 2022. Exclusion criteria were: (a) severe motion artifacts on CCTA, (b) contraindication to contrast medium injection, (c) previous coronary artery stenting or bypass grafting, and (d) BMI > 30 kg/m^2^. Patients exhibiting a heart rate (HR) > 75 bpm received treatment with intravenous beta-blocker (metoprolol tartrate, 5 mg).

The required sample size was calculated using G*Power software (version 3.1.9.7), aiming for a statistical power of 0.80 and a significance level of 0.05. Considering a comparison of three different groups of patients, assuming a medium effect size (*f* = 0.25), the calculation indicated that 84 participants per group were needed, resulting in a total sample size of 252 participants. Participants were randomly assigned (1:1 ratio) into three groups: group A, group B, and group C.

### Image acquisition

CCTA examinations were conducted utilizing a 64-slice CT (GE Revolution EVO CT Scanner, GE Healthcare) in the cranio-caudal direction. A retrospective electrocardiogram (ECG)-gated protocol was employed, featuring the following scan parameters: detector collimation of 0.625 mm × 64, gantry rotation time of 0.6 s, spiral pitch dynamically adjusted based on heart rate, varying from 0.16 to 0.30, and a matrix size of 512 × 512 pixels.

Automatic exposure control was implemented with a range of 150–480 mAs in all groups while tube voltage and contrast medium volume varied according to the three groups. The duration of contrast medium injection was determined to be the sum of scan time plus the minimum diagnostic delay of the CT scan (6 s).

For all CCTA, a non-ionic high-iodine concentration contrast medium (Iomeron 400 mgI/mL, Bracco Imaging) was administered intravenously via the antecubital vein, with an automated triple-syringe power injector (MEDRAD® Centargo CT Injection System; Bayer AG), followed by a 30 mL saline chaser bolus injected at an identical flow rate.

CCTA of group A was acquired with 100 kVp tube voltage and an iodine delivery rate (IDR) of 1.8 g/s. CCTA of group B and group C were both acquired with 80kVp tube voltage and an IDR of 1.4 g/s and 1.2 g/s, respectively. Consequently, to achieve the established IDR, the flow rates were 4.5 mL/s for group A, 3.5 mL/s for group B, and 3 mL/s for group C, respectively.

The scan delay was established using a bolus-tracking software program (SmartPrep, GE Healthcare): CCTA acquisition started as soon as the trigger attenuation threshold (100 HU) was reached into a region-of-interest (ROI) positioned in the ascendent aorta at the level of the pulmonary arteries, ensuring the minimum diagnostic delay.

### Image reconstruction

Each examination was reconstructed at a slice thickness of 0.625 mm. CCTA in group A was reconstructed by ASIR-V at a strength level of 50%, while CCTA of groups B and C were reconstructed by DLIR-H.

### Objective image quality analysis

Quantitative measurements were conducted by a radiologist with 10 years of expertise in cardiovascular imaging, on a dedicated workstation (Advantage Workstation 4.7, GE Healthcare) for each CCTA examination.

An ROI was drawn, on axial slices, in the left pectoral muscle, ascending aorta, left main artery (LM), left anterior descending artery (LAD), left circumflex artery (LCX), and right coronary artery (RCA), carefully avoiding the inclusion of vessel walls and atherosclerotic plaques. For each coronary artery, the ROI was placed in the proximal, medium, and distal segments. Image noise was defined as the standard deviation (SD) of the ROI placed in the pectoral muscle.

All ROIs were positioned three times, and measurements were subsequently averaged to mitigate potential inaccuracies.

The signal-to-noise ratio (SNR) was determined using the following formula:

$${{\rm{SNR}}}=\frac{{{\rm{HU}}}_{{\rm{artery}}}}{{{\rm{SD}}}_{{\rm{muscle}}}}$$

Contrast-to-noise ratio (CNR) was calculated as follows:

$${{\rm{CNR}}}=\frac{{{\rm{HU}}}_{{\rm{artery}}}-{{\rm{HU}}}_{{\rm{muscle}}}}{{{\rm{SD}}}_{{\rm{muscle}}}}$$

### Subjective image quality analysis

Two additional radiologists with 10 years and 5 years of experience in CCTA, blinded to the reconstruction protocol were enlisted to assess the subjective image quality of all images using a 5-point Likert scale, specifically: 1, poor; 2, adequate; 3, moderate; 4, good; and 5, excellent image quality. To mitigate recall bias, the images were assessed in a random order. Standard window settings (width, 1200 HU; level, 240 HU) were initially applied, but adjustments were allowed to accommodate the readers’ preferences. Ambient lighting condition was kept constant at approximately 35–40 lux.

### Radiation dose and contrast dose

For each participant, the dose–length product (DLP) in milli-gray-centimeter (mGy·cm) was recorded. Effective dose (ED) in millisievert (mSv) was estimated by multiplying the DLP in mGy·cm by a conversion coefficient of 0.014 mSv/(mGy·cm). Contrast medium volume was also recorded.

### Statistical analysis

Continuous data were expressed as mean ± SD if normally distributed, nonparametric data were expressed as median and interquartile range (IQR).

Normally distributed continuous data were compared using Student’s *t*-test. The sex ratio and the BMI ≥ 25 kg/m^2^ ratio were compared with the Chi-square test. Objective image quality parameters between the three groups were analyzed by one-way ANOVA test.

The Weighted Cohens’ kappa test was used to test the inter-rater agreement for the qualitative analysis of image quality with kappa values: > 0.80 indicating almost perfect agreement, 0.60–0.79 substantial, 0.40–0.59 moderate, 0.21–0.39 fair, and < 0.20, none to slight agreement. A *p* value < 0.05 was considered statistically significant. Statistical analyses were performed with MedCalc, version 20.215 (MedCalc Software Ltd).

## Results

### Patient population

**Fig. 1:**
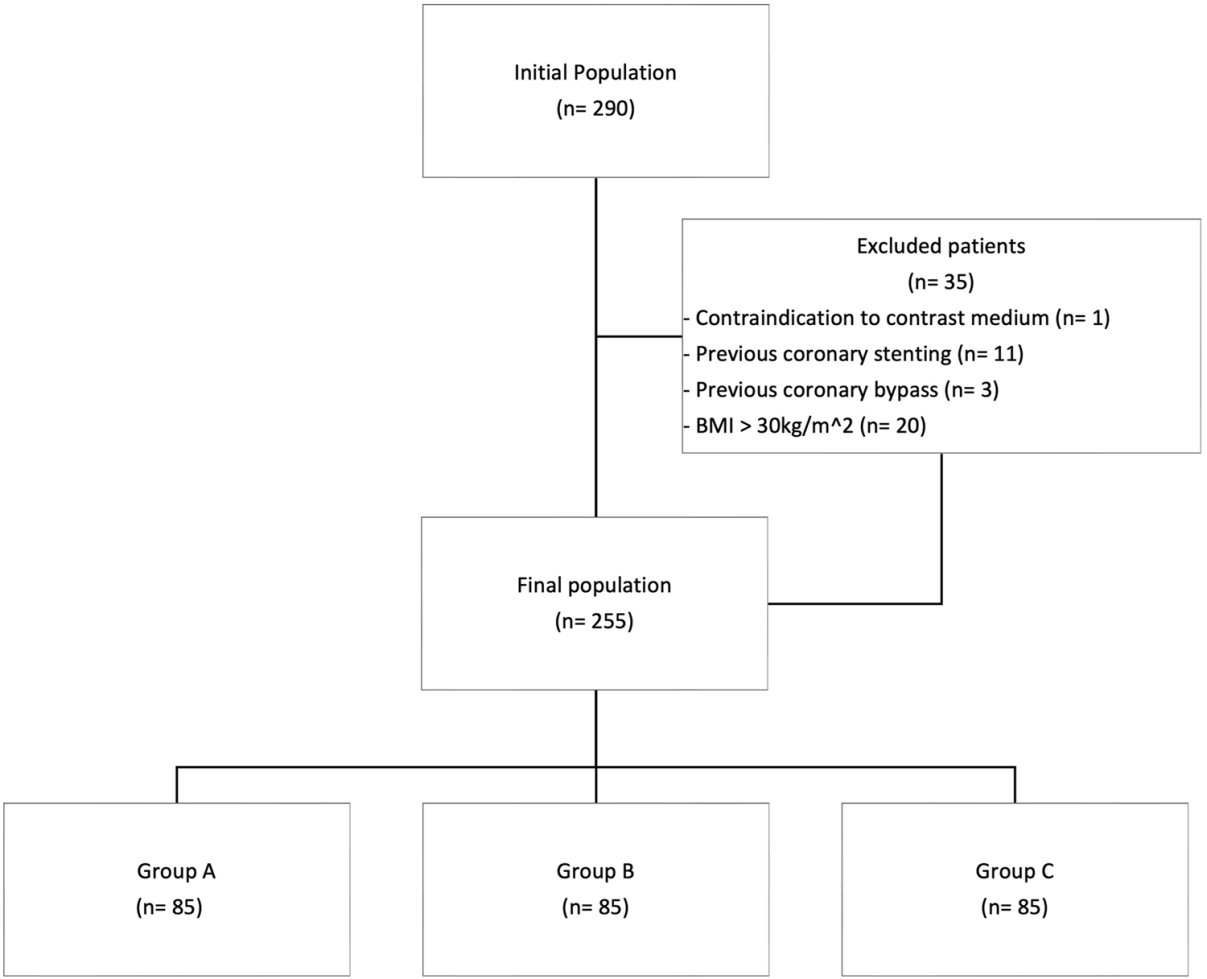
Flow diagram of patient recruitment. BMI, body mass index; DLIR-H, high strength deep learning iterative reconstruction; IDR, iodine delivery rate; ASIR, adaptive statistical iterative reconstruction

Out of an initial population of 290 individuals, 35 participants were excluded from the analysis due to contraindication to contrast medium (*n* = 1), previous coronary stenting (*n* = 11), previous coronary bypass (*n* = 3), and BMI > 30 kg/m^2^ (*n* = 20). Thus, the final population consisted of 255 patients, 85 patients in each group, Fig. 1. Patient age did not vary significantly across the three groups (group A: 64.7 ± 10 years; group B: 64.9 ± 11 years.; group C: 63.9 ± 10 years; all *p* ≥ 0.516). BMI ranged from 26 ± 3.1 kg/m^2^ in group A to 25 ± 3.2 kg/m^2^ in group C, with a slightly significant difference between these two groups (*p* = 0.040), while other comparisons returned comparable values (all *p* ≥ 302). Detailed results are reported in Table 1.

**Table 1:** Patient characteristics

	Groups			*p* value		
	Group A	Group B	Group C	A vs B	A vs C	B vs C
Population^a^	85	85	85			
Male/female ratio	64/21	51/34	46/39	0.087	0.019	0.516
Age (years)^b^	64.7 ± 10	64.95 ± 11	63.9 ± 10	0.878	0.605	0.516
BMI (kg/m^2^)^b^	26 ± 3.1	25.5 ± 3.2	25 ± 3.2	0.302	0.040	0.309
≥ 25^a^	53	48	40	0.516	0.090	0.294
< 25^a^	32	37	45	0.494	0.083	0.290
Heart rate (bpm)^b^	61 ± 8.5	59 ± 9.1	62 ± 6.3	0.140	0.384	0.013

*BMI* body mass index, *bpm* beats per minute

^a^ Data are frequency

^b^ Data are mean ± SD

### Objective image quality

**Fig. 2:**
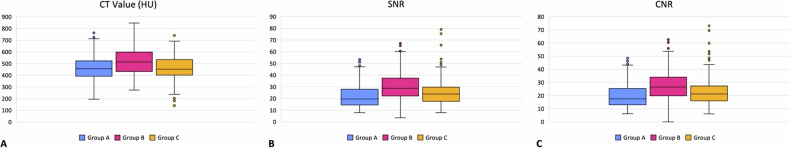
Box-and-whisker plots for quantitative image quality show the distribution of coronary arteries attenuation values (**A**), SNR (**B**), and CNR (**C**) of groups A, B, and C. Boxes represent the middle 50% of the data, solid lines represent the median, and whiskers represent minimum and maximum values. Group B yielded significantly higher attenuation values, SNR, and CNR (all *p* < 0.001)

Comprehensive objective image quality results are reported in Table 2 and depicted in Fig. 2, with a total of 6120 datasets subjected to analysis.

**Table 2:** Objective image quality scores

	Groups			*p* value		
	Group A	Group B	Group C	A vs B	A vs C	B vs C
Noise^a^	23.6 ± 6.7	18.5 ± 5.4	20.7 ± 7.3	< 0.001	0.008	0.027
CT value^a^						
Average	461.1 ± 28.9	517.3 ± 26.3	460 ± 24.3	< 0.001	0.788	< 0.001
Aorta	488.9 ± 98.1	545.1 ± 105.9	487.2 ± 97	< 0.001	0.909	< 0.001
LM	469.7 ± 103.9	523 ± 125.3	468.5 ± 89.3	0.003	0.936	0.001
LAD	441.8 ± 104.9	512.4 ± 105.3	444.9 ± 91.9	< 0.001	0.838	< 0.001
LCX	447.8 ± 100.8	499.1 ± 108.9	442.6 ± 93.2	0.002	0.727	< 0.001
RCA	457.6 ± 113.4	506.9 ± 130.3	456.9 ± 100.2	0.009	0.966	0.006
SNR						
Average	21.6 ± 9.2	30.5 ± 11.5	24.4 ± 12.1	< 0.001	0.091	< 0.001
Aorta	22.9 ± 9.7	32.1 ± 11.6	24.3 ± 20.9	< 0.001	0.576	0.003
LM	21.9 ± 9.2	30.8 ± 11.9	25.2 ± 10.1	< 0.001	0.027	0.001
LAD	20.8 ± 9.1	30.2 ± 11.1	23.9 ± 9.7	< 0.001	0.033	< 0.001
LCX	20.9 ± 8.7	29.4 ± 10.8	23.8 ± 9.8	< 0.001	0.043	< 0.001
RCA	21.5 ± 9.6	30 ± 12.1	24.6 ± 10.4	< 0.001	< 0.001	0.002
CNR						
Average	19.6 ± 8.7	27.8 ± 11	22.5 ± 9.5	< 0.001	0.039	0.001
Aorta	20.9 ± 9.1	29.4 ± 11.1	23.7 ± 9.9	< 0.001	0.056	< 0.001
LM	19.9 ± 8.7	28.1 ± 11.5	22.8 ± 9.5	< 0.001	0.039	0.001
LAD	18.7 ± 8.6	27.5 ± 10.6	21.6 ± 9.1	< 0.001	0.003	< 0.001
LCX	18.6 ± 8.2	26.7 ± 10.3	21.4 ± 9.3	< 0.001	0.038	< 0.001
RCA	19.4 ± 9.0	27.3 ± 11.6	22.3 ± 9.9	< 0.001	0.047	0.003

Data are mean ± SD. Parametric continuous data are expressed means ± SD

*CNR* contrast-to-noise ratio, *LM* left main, *LAD* left anterior descending branch, *LCX* left circumflex branch, *SNR* signal-to-noise ratio, *RCA* right coronary artery

^a^ Hounsfield units

Group B yielded higher attenuation values (517.3 ± 26.3 HU) than group A and C (461.1 ± 28.9 HU and 460 ± 24.3 HU, respectively; *p* ≤ 0.001); no significant differences were found between group A and C (*p* = 0.788). Similarly, group B yielded also lower noise (18.5 ± 5.4) than group A and C (23.6 ± 6.7 and 20.7 ± 7.3, respectively; all *p* < 0.001).

Group B yielded also higher overall SNR (30.5 ± 11.5) and CNR (27.8 ± 11) compared to group A (21.6 ± 9.2 and 19.6 ± 8.7) and group C (24.4 ± 12.1 and 22.5 ± 9.5, all *p* ≤ 0.001). Group C achieved higher CNR than group A (22.5 ± 9.5 vs 19.6 ± 8.7; *p* = 0.039), while the SNR of the two groups was comparable (24.4 ± 12.1 vs 21.6 ± 9.2; *p* = 0.091).

### Subjective image quality

**Fig. 3:**
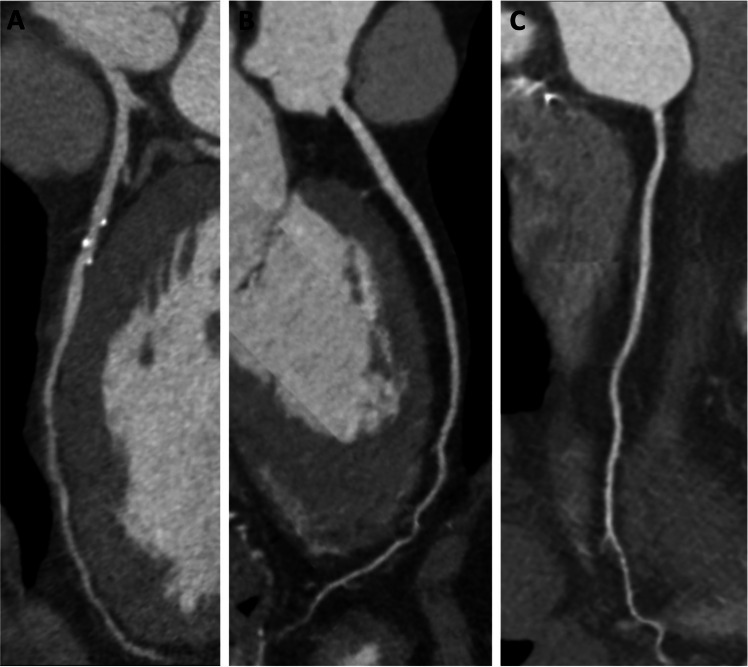
Curved multiplanar reformations depicting LAD arteries of a 53-year-old man assigned to group A (**A**), a 69-year-old woman assigned to group B (**B**), and a 63-year-old woman assigned to group C (**C**), respectively. All studies were deemed diagnostic. Group B (score: 4, interquartile range: 3–4) outperformed both group C (score: 3, interquartile range: 3–3) and group A (score: 3, interquartile range: 3–4; all *p* < 0.001); no statistically significant differences have been found between group A and C (*p* = 0.338)

All CCTA images were deemed diagnostic (Fig. 3). Group B (4, IQR [3–4]) outperformed both group C (3, IQR [3–3]) and group A (3, IQR [3–4]; all *p* < 0.001), while no statistically significant differences were observed between group A and C (*p* = 0.338). The inter-rater agreement between the two readers was almost perfect in all groups (group A, κ = 0.91; group B, κ = 0.845; and group C, κ = 0.888).

### Radiation dose and contrast dose

Groups B and C yielded 42% and 41.2% ED reduction (2.36 ± 0.9 mSv and 2.39 ± 0.6 mSv respectively) compared to group A (4.07 ± 1.2 mSv; *p* < 0.001). Moreover, no significant differences were observed between group B and group C (*p* = 0.79).

The contrast medium dose in group C (44.8 ± 4.4 mL) was sensibly lower than group A (57.7 ± 6.2 mL) and B (50.4 ± 4.3 mL; all *p* < 0.001). Contrast medium dose between groups A and B was also significantly different (*p* < 0.001). The complete results detailing radiation dose and contrast medium dosage are provided in Table 3.

**Table 3:** Contrast dose and radiation dose comparison among the three groups

	Group A	Group B	Group C	*p* value		
				A vs B	A vs C	B vs C
DLP (mGy·cm)	290.9 ± 84.8	168.9 ± 63.2	170.49 ± 40.1	< 0.001	< 0.001	0.84
ED (mSv)	4.07 ± 1.2	2.36 ± 0.9	2.39 ± 0.6	< 0.001	< 0.001	0.79
Contrast medium volume (mL)	57.7 ± 6.2	50.4 ± 4.3	44.8 ± 4.4	< 0.001	< 0.001	< 0.001

Data are mean ± SD

*DLP* dose–length product, *ED* effective dose

## Discussion

In this investigation, we demonstrated the effectiveness of the DLIR-H algorithm applied to 80-kVp CCTA in reducing radiation dose and contrast medium dose (“double-low” condition) without compromising image quality in patients with BMI < 30 kg/m^2^, compared with the standard 100 kVp protocol coupled with ASiR-V.

Several studies have proposed imaging protocols designed to minimize the impact of CCTA by seeking a compromise between reducing radiation dose and preserving diagnostic image quality. Among the multitude of strategies, many were linked to the use of prospective ECG-triggering, high-pitch acquisition, and low-tube voltage techniques [15–20]. Reducing the X-ray tube voltage peak is an effective method to reduce radiation dose, since the latter varies in proportion to the square of tube voltage [9, 21]. Studies by Zhang et al [19] and Pflederer et al [22] have demonstrated significant radiation dose reduction with 100-kVp protocols, particularly in patients with low BMI and low body weight. Similarly, Oda and coworkers [23], achieved substantial radiation dose reductions with an 80-kVp CCTA protocol in patients with BMI < 25 kg/m^2^, with no detrimental effect on image quality. Nevertheless, none of these studies coupled low-kVp protocols with contrast medium reduction. On the other hand, high-iodine concentration contrast media further facilitate low-voltage scanning protocols, allowing for a reduction in the volume of the administered contrast medium, and improving image quality in critical scenarios, such as in obese patients [7].

The double-low strategy in CCTA was implemented by Komatsu et al and Cao et al in patients with low coronary artery calcium burden and BMI < 26 kg/m^2^ and < 23 kg/m^2^, respectively [24, 25]. Zhang LJ et al and Feng et al both obtained diagnostic image quality under the double-low condition using 30 mL of contrast medium in a 70-kVp protocol, although limited to patients with BMI < 26 kg/m^2^ [21, 26].

Low-kVp scanning protocols have been mostly investigated on low-BMI individuals due to the double effect related to low-kVp scans: contrast medium attenuation is maximized owing to the X-ray absorption characteristics of iodine; however, the use of low-kVp settings concurrently leads to an increased image noise, especially in individuals with high BMI [9]. Nonetheless, BMI is a major cardiovascular risk factor and, in patients with established coronary atherosclerosis, is associated with acute coronary syndromes [27]. Consequently, achieving diagnostic image quality with a low-voltage protocol poses a significant technical challenge [28].

Iterative reconstruction (IR) algorithms have been instrumental in minimizing image noise in low-dose protocols and currently represent the standard reconstruction algorithm in most CT examinations [29, 30]. However, In clinical practice, these algorithms are typically applied at medium strength levels to avoid over-smoothing and “plastic-looking” images, especially along the borders of blood vessels [31].

Artificial intelligence (AI) has witnessed substantial growth in recent years, particularly in the field of image reconstruction. Specifically, DLIR algorithms, based on deep neural networks, consist of numerous layers of mathematical equations designed to determine the optimal solution to a given problem [32, 33]. Consequently, DLIR has emerged as a powerful reconstruction method, providing effective noise reduction and enhanced image quality. Unlike iterative reconstruction (IR) techniques, DLIR is less sensitive to variations in radiation dose, ensuring consistent image quality across different exposure levels. Additionally, DLIR maintains a negligible impact on image texture, ultimately translating into better subjective image quality [10–12]. Studies by Li et al [14] and Sun et al [34] have demonstrated the efficacy of DLIR-H in reducing contrast medium and radiation dose while improving image quality in CCTA protocols, in patients with BMI < 26 kg/m² and in pediatric populations, respectively. Our investigation validates these previous findings by expanding the study population up to BMI < 30 kg/m², highlighting the potential broader applicability of DLIR-H in a larger group of individuals more susceptible to CAD.

A low IDR results in contrast medium reduction, which in turn negatively impacts vascular attenuation and objective image quality [35]; nevertheless, this drawback may be successfully counterbalanced by DLIR-H: we demonstrated that groups B and C succeeded in achieving the “double-low” goal. Additionally, group B yielded the highest overall image quality. Group C, on the other hand, despite requiring the lowest amount of contrast medium, was qualitatively less performing than group B, which appears to be the best solution.

Noteworthily, the advent of the novel photon counting detector (PCD)-CT technology could represent a further turning point in the search for the optimal CCTA study protocol. PCD-CT is currently under active investigation and has demonstrated higher image quality than conventional energy-integrating detector CT [36]. Additionally, PCD-CT enables a significant reduction of contrast media volume at CCTA using low-energy VMI, up to 50% in phantom investigations and 40% in clinical settings, while maintaining diagnostic image quality [37–39]. The applicability of a double-low protocol with image quality assessment by the novel PCD-CT represents a very interesting topic worthy of further investigation in future studies.

Our study has several limitations. First, only 20 patients, accounting for 8%, underwent an invasive coronary angiography. Therefore, diagnostic accuracy was tested only in these patients. Second, patients with coronary stents were excluded from our analysis; the assessment of the stents with the DLIR represents another interesting field and, therefore, deserves a specific study. Third, our results are vendor-specific, so the generalizability of our findings to other DLIR algorithms from different vendors may be limited. Lastly, the objective evaluation of image quality was performed by a single reader, albeit with extensive experience in cardiovascular imaging.

To conclude, DLIR-H applied to CCTA allows the use of a double-low dose protocol, consisting of an X-ray tube output of 80 kVp coupled with an IDR of 1.4 g/s, obtaining better images than the conventional CCTA protocol in patients with BMI < 30 kg/m^2^.

## Abbreviations

ASIR: Adaptive statistical iterative reconstruction
BMI: Body mass index
CCTA: Coronary CT angiography
CNR: Contrast-to-noise ratio
DLIR-H: High-strength deep learning image reconstructions
DLP: Dose–length product
ED: Effective dose
IDR: Iodine delivery rate
ROI: Region-of-interest
SNR: Signal-to-noise ratio
